# Remarkable Deformity of a Human Fœtus

**Published:** 1845-09

**Authors:** 


### REMARKABE DEFORMITY IN A HUMAN FŒTUS.

In a letter to the Editors, Dr. D. G. Gregory, of Platte county, in Missouri, gives the following account of a fœtus singularly deformed, the birth of which he attended.

GENTLEMEN:—

The following case presents so many deformities meeting in the same subject, that I think it is worthy of being placed upon record. If you are of the same opinion you may give it a place in your Journal.

July 22d, 1845—I was requested to see Mrs. G. in labor with her sixth child. When I arrived the labor had been in progress nearly 24 hours; the uterine contractions were strong and frequent; an examination, per vaginam, discovered the fœtus engaged in the inferior strait of the pelvis, but so uncommon was the shape, that I could not ascertain the presentation, and was for sometime at a loss to know what course to pursue; but as there seemed to be no obstruction to the delivery, I concluded to await the rupture of the membranes, which soon took place, when one hand, and then the other presented at the os externum. This led me to search for the head, but no satisfactory discovery could be made. In this state of things I concluded to let nature take her own course, and was much relieved at finding that the delivery was soon to be accomplished. The following was the appearance of the unsightly object which presented itself in the shape of a female fœtus:

*Head.*—The bones of the cranium were entirely wanting, and a fleshy mass, of the color of coagulated blood, protruded at the crown.

*Face.*—The forehead was very short; there were deep fissures where the eyes should have been, but there were no eyes, eye-lashes, or eye-brows; there was the shape of a nose, which was broad, and seemed to grow on a level with the surrounding parts of the face. There were openings for the ears, but the external ear was very small and ill shaped. The upper lip and roof of the mouth were cleft, as in the worst form of hare-lip; the under lip and chin were well formed, and of the usual size. The tongue adhered closely to the lower jaw.

*Superior extremities.*—The arms were very short, especially the forearms; hands very short, with five fingers and a thumb on each.

The thorax was well formed and capacious; the abdomen enormously large, and unsightly.

The external organs of generation were very imperfectly developed. The thighs were short, and the legs equally so, and flexed upon the thighs; the feet were crooked, presenting a form of club-foot, with five small toes, and the first, or great toe *double*, making seven toes on each foot.

The length of the fœtus was 15 inches; it was born alive, gasped once or twice after birth, but expired almost instantly.

One object I have had in offering the foregoing communication is to obtain from you, or some of your numerous correspondents, information respecting the cause of such monstrous births, if indeed any cause can be assigned for them. What are the conjectures of physiologists on the subject? Is it for want of proper nourishment from the mother during gestation, or is it from malformation, or disease of the uterus, or ovaries? or owing to the want of proper health and energy in the generative functions of either of the parents?

The mother, in this case, was healthy and well formed, having borne three healthy children, which are still living, and three that have died, two of the six being deformed. The father is unhealthy, and has been so for a number of years.

July 28th, 1845.

